# Storage stabilization of microbes for biotechnology: An annotated selection of World Wide Web sites relevant to the topics in microbial biotechnology

**DOI:** 10.1111/1751-7915.13888

**Published:** 2021-07-26

**Authors:** Lawrence P. Wackett

**Affiliations:** ^1^ Department of Biochemistry Molecular Biology & Biophysics, BioTechnology Institute University of Minnesota St Paul MN 55108 USA

## Storage stability of microbes for culture collections

https://pubmed.ncbi.nlm.nih.gov/25773973/

In culture collections, microbes are typically stored after freeze‐drying. This study examined microorganisms known to be recalcitrant to storage and examined drying conditions that would lead to the best outcomes.

## Storage of bacteria for optimum viability

https://www.thermofisher.com/us/en/home/industrial/microbiology/microbiology‐learning‐center/storing‐bacterial‐samples‐optimal‐viability.html

This commercial website provides a good overview of different bacterial storage methods, the temperatures they require and the effects on bacteria generally.

## Cryo‐storage of algae for biotechnology

https://www.nature.com/articles/s41598‐019‐38588‐6

Increasingly, algal storage is becoming industrially relevant. This study used *Chlorella vulgaris* as an example and showed that ultra‐low temperature and nitrogen control were important factors in maintaining viability.

## Probiotics stabilization

https://www.intechopen.com/books/probiotics/different‐methods‐of‐probiotics‐stabilization

This report describes a wide range of methods for preparation and storage of probiotic bacterial starter cultures.

## Formulation and stabilization of an *Arthrobacter*

https://link.springer.com/article/10.1007/s00253‐017‐8706‐6

This study examined *Arthrobacter chlorophenolicus* A6 that biodegrades chlorophenols. They examined methods like air‐drying in media such as vermiculite, to store bacteria for use in bioremediation.

## Storage of microbial communities in stool samples

https://journals.plos.org/plosone/article?id=10.1371/journal.pone.0227486

Given the increasing interest in metagenomic studies of gut microbiomes, accurate and reproducible comparative data often requires maintaining microbial viability while storing stool samples. This study examined storage conditions in this context.

## Stabilizing gut microbiome DNA

https://onlinelibrary.wiley.com/doi/full/10.1002/mbo3.1046

This study describes the use of a guanidine thiocyanate based medium to stabilize microbial DNA in gut microbiome sample for shipping, storage and later sequencing.

## Mechanisms of trehalose protection during bacteria desiccation storage

https://link.springer.com/article/10.1007/s00294‐019‐01036‐z

Trehalose has been known to prevent cellular protein damage by acting as a chemical chaperone, but it might also diminish protein acetylation and glycation.

## Storage of non‐sporulating bacterial inoculants

https://sfamjournals.onlinelibrary.wiley.com/doi/10.1111/1751‐7915.12880

This review article discusses methods for maintaining high viability in microbial inoculant cultures for use in agriculture.

## Stabilizing bacteria for seed coating

https://patents.google.com/patent/US8011132B2/en

Numerous genera of bacteria have been shown to provide benefits to plant growth and those include: *Rhizobium, Bradyrhizobium, Bacillus, Azotobacter* and *Azospirillum* species. This patent deals with the storage of these types of bacteria in liquid inoculants on seeds.

## Life and death of dried prokaryotes

http://www.esalq.usp.br/lepse/imgs/conteudo_thumb/Life‐and‐death‐of‐dried‐prokaryotes.pdf

This classic paper focuses on the underlying molecular mechanisms of desiccation resistance and sensitivity in prokaryotes.

## Arctic and Antarctic desiccation‐tolerant microorganisms

https://www.biorxiv.org/content/10.1101/2021.02.06.430066v1.full

This preprint deals with desiccation tolerant bacteria in cold adapted dry environments where water is in a solid state during almost the entire year.

